# Intrasession repeatability of pupil size measurements under different light levels provided by a multidiagnostic device in healthy eyes

**DOI:** 10.1186/s12886-020-01625-4

**Published:** 2020-08-31

**Authors:** David P. Piñero, Dolores de Fez, Inmaculada Cabezos, Alberto López-Navarro, María T. Caballero, Vicente J. Camps

**Affiliations:** 1 Group of Optics and Visual Perception, Department of Optics, Pharmacology and Anatomy, University of Alicante, Crta San Vicente del Raspeig s/n 03016, San Vicente del Raspeig, Alicante, Spain; 2 Optometry Clinic, Prevention Service of the University of Alicante, Alicante, Spain

**Keywords:** Scheimpflug imaging, VX120 system, Pupillometry, Pupil diameter, Scotopic, Mesopic, Photopic

## Abstract

**Background:**

The measurement of the pupillary function is an indispensable test in some eye examinations, being necessary the evaluation of the precision of instruments performing such measures. The aim of this study was to evaluate the intrasession repeatability of pupil size measurements provided by a multidiagnostic platform in a large sample of healthy eyes.

**Methods:**

This prospective study enrolled 100 healthy eyes of 100 patients, with ages ranging from 23 to 65 years old. Repeated pupil size measures under photopic (P, 220 lx), mesopic (M, 160 lx), low mesopic (L, 70 lx), and scotopic conditions (S, 1 lx) were obtained with the VX120 system (Visionix-Luneau Technologies, Chartres, France) after a complete eye exam. Likewise, pupil size was also measured once in the fellow eye in a total of 75 eyes. The level of intrasession variability as well as differences between fellow eyes were evaluated.

**Results:**

Most of differences between repeated measures did not exceed 0.5 mm (82% of S and 100% of P below this value). No significant differences between these repeated measures were found for S (*p* = 0.099) and L (*p* = 0.751). However, statistically significant differences were found between repeated measures for M (*p* = 0.002) and P (*p* = 0.003). The analysis of clinical relevance of differences between pairs (Passing-Bablok) only confirmed the clinical relevance of differences between the first and second repeated measurement of M. Concerning the comparative analysis between fellow eyes, no statistically significant differences in pupil size were found between right and left eyes in any light condition evaluated (*p* ≥ 0.227).

**Conclusions:**

The VX120 system can provide consistent measurements of pupil size under scotopic, low mesopic and photopic conditions, with a relative limitation under mesopic conditions.

## Background

The evaluation of the pupillary function is an indispensable test in some eye examinations, especially in those related to neurological disorders [1] and screening for refractive surgery [2]. Several devices have been developed to analyse the pupillary function in the clinical practice [2–10], but not all of them have been validated. Indeed, the scientific literature on validation of pupillometers is scarce and with some limitations. Among these limitations, the most relevant are the use of small samples of subjects used for the validations, the great variability among studies in the light conditions used during the measurements, and the performance of comparative studies between devices, but not considering an intrasession repeatability analysis [2–10]. For this reason, more validation studies of new pupillometers are required to ensure their real clinical usefulness.

A new multidiagnostic platform, the VX120 system (Visionix-Luneau Technologies, Chartres, France), has been recently developed allowing the clinician to obtain automatic measurements of corneal topography, anterior segment anatomical parameters, corneal, internal and ocular aberrations, intraocular pressure (IOP), and pupil diameter under different light conditions. Different studies have been conducted to validate some of these measurements provided by this multidiagnostic system in healthy eyes [11–13], but there is not specific validation of its pupil size measurements. The aim of the current study was to evaluate the intrasession repeatability of pupil size measurements provided by this multidiagnostic platform in a large sample of healthy eyes.

## Methods

### Patients

This prospective study of evaluation of a technology included a total of 100 healthy eyes of 100 patients, with ages ranging from 23 to 65 years old. Repeated pupil size measures were obtained with the VX120 system in one eye from each subject that was selected randomly according to a random number sequence (dichotomic sequence, 0 and 1) with the aim of avoiding the potential interference of the correlation that often exists between the two eyes of the same person. Likewise, the measurement of pupil size was also obtained once in the fellow eye in a total of 75 eyes. The study was conducted at the Optometry Clinic of the University of Alicante. Before examinations, all patients were informed about the study and signed an informed consent in accordance with the tenets of the Helsinki Declaration. This study was approved by the Ethics Committee of the University of Alicante (Spain).

Inclusion criteria were eyes without active ocular pathology, age of more than 18 years, and the presence of a refractive error between + 5.00 and − 10.00 D. Exclusion criteria for the study included systemic pathological conditions at the moment of examination, previous ocular surgery, neurological disorders, glaucoma, pseudophakia, and anisocoria (more than 0.5 mm of difference between right and left eye for the measurements obtained under the four light level conditions simulated by the multidiagnostic system used).

### Examination protocol

A complete eye exam was performed in all patients including measurement of uncorrected (UDVA) and corrected distance visual acuity (CDVA), manifest refraction, fundus evaluation, and air tonometry, pupillometry and anterior segment analysis with the VX120 system (Visionix-Luneau Technologies, Chartres, France). The same experienced examiner (ALN) performed all pupil size measurements in a dark room after a period of 2 min of patient adaptation. Specifically, the repeatability of the measurement of the following pupil sizes was evaluated: scotopic pupil diameter (S, 1 lx, only infrared light), low mesopic pupil diameter (L, 70 lx), mesopic pupil diameter (M, 160 lx), and photopic pupil diameter (P, 220 lx).

### The VX120 system

The VX120 system is a multidiagnostic platform that combines several technologies to provide an integral examination of the eye. Specifically, a Placido-disk corneal topographer, a Scheimpflug camera, a Hartmann-Shack aberrometer, an infrared pupillometer and an air tonometer are combined in the same platform. The pupillometer module uses a camera to obtain the images of the pupil and to take the measurements of the pupil size. The system can measure pupil diameters of 2 mm or more, using a point light as a fixation stimulus and a variable ambient illumination (455 nm). The same automatic sequence of pupil size measurements is always performed by the instrument:

1. Scotopic (S, 1 lx, only infrared light)
2. Low mesopic (L, 70 lx)
3. Mesopic (M, 160 lx)
4. Photopic (P, 220 lx).

### Statistical analysis

The software SPSS version 15.0 for Windows (SPSS, Chicago, Illinois, USA) was used to perform the statistical analysis of the data obtained in this study. First, the Kolmogorov-Smirnov test was used to confirm if the pupil size data distributions did follow or not a Normal distribution. Only the repeated pupil size measurements under scotopic conditions followed a normal distribution (*p* = 0.20), while the rest of measurements were not normally distributed. For the analysis of the scotopic pupil measurements (S), differences between repeated measurements were evaluated with an analysis of variance (ANOVA) of repeated measurements, with post-hoc comparison between pairs using the paired Student t test with the Bonferroni correction. Likewise, the within-subject standard deviation (S_w_) of repeated measurements and the intraclass correlation coefficient (ICC) were also calculated. For photopic (P) and mesopic (M and L) measurements, the Friedman test was used to assess the significance of differences between repeated measurements, with post-hoc comparison between pairs using the Wilcoxon test with the Bonferroni correction. In those cases where significant differences were found, the interchangeability of repeated measurements was evaluated using the Passing-Bablok analysis. It should be considered that the Bland and Altman method could not be used because these data were not normally distributed. Finally, a comparison of right and left eye pupil size measurements was performed in a subgroup of subjects in which a bilateral measurement was obtained (75 patients). For this comparison, the paired Student t test and Wilcoxon test were used for normally and not normally distributed data, respectively.

## Results

### Intrasession repeatability

This analysis involved 100 eyes of 100 patients in which three consecutive measurements of pupil diameter were obtained. Table 1 shows the results of each repeated pupil size measurement under the four illumination conditions used. As shown, median and standard deviations of each repeated measurement under each light condition were similar. Specifically, there was a mean decrease in S pupil diameter in the second measurement (0.2 mm) and an increase in the third, while the fluctuation between repeated measurements under L, M and P levels was below 0.1 mm. Regarding the difference in the amplitude of the range in each repeated measurement, variations between 0.2 and 0.3 mm were found.

**Table 1:** Mean, median, standard deviation (SD), variance and range for each repeated pupillometric measurement (100 patients) under the four illumination conditions evaluated. S: Scotopic level, L: Low level, M: mesopic level, P: Photopic level

	S1	S2	S3	L1	L2	L3	M1	M2	M3	P1	P2	P3
Mean	5.38	5.32	5.33	3.23	3.24	3.23	2.88	2.96	2.93	2.57	2.60	2.60
Median	5.50	5.30	5.35	3.20	3.20	3.10	2.80	2.90	2.85	2.60	2.55	2.60
SD	0.96	0.87	0.90	0.65	0.57	0.59	0.47	0.47	0.49	0.38	0.40	0.38
Variance	0.93	0.76	0.81	0.43	0.33	0.35	0.22	0.22	0.24	0.15	0.16	0.14
Range	4.00	3.80	3.70	2.80	2.90	2.70	2.60	2.90	2.70	2.30	2.50	2.40

Table 2 shows the mean, median, standard deviation (S_w_) and the maximum difference between repeated measurements under each light condition. Although the maximum difference among the three repeated measurements for each of the four illumination levels was around 1 mm (except for P, which was 0.5 mm), most differences did not exceed 0.5 mm (between 82% of S and 100% of P measures were below this value).

**Table 2:** Median, standard deviation (S_w_) and maximum difference between repeated measurements under each light condition evaluated. Likewise, the percentage of eyes with a range of variation of repeated measurements of 0.5 mm or lower was also provided

	S	L	M	P
Median (mm)	5.3	3.2	2.9	2.6
S_w_ (mm)	0.2	0.1	0.1	0.1
Max difference (mm)	1.0	0.9	1.0	0.5
Max difference < =0.5 mm (%)	82	97	96	100

For S pupil size measurements, differences between repeated measurements were not statistically significant (*p* = 0.099). The ICC was 0.97, which is consistent with an excellent consistency (ICC > 0.91). The analysis of the correlations between the three pairs of measures confirmed that all of them were high (r > 0.94, *p* < 0.01).

Regarding the influence of age on the repeatability of pupil size measurements, very poor correlations were found between age and the S_w_ values associated to the different pupil size measures (S, r = − 0.047, *p* = 0.645; L, r = − 0.150, *p* = 0.136; M, r = − 0.004, *p* = 0.971; P, r = − 0.259, *p* = 0.009).

**Fig. 1:**
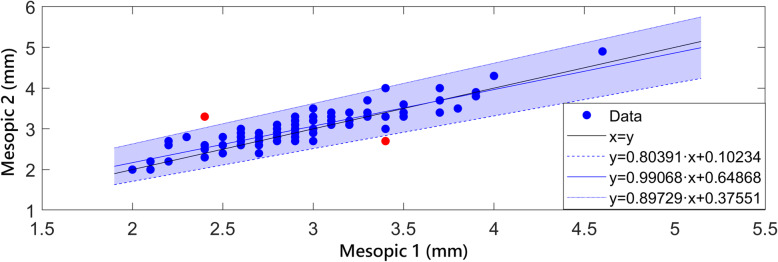
Passing-Bablok interchangeability analysis of the first and second measurements of pupil size under mesopic conditions. The confidence interval of x-intercept (coloured area) and slope of the adjusted lines does not include the values of 0 and 1, respectively

**Fig. 2:**
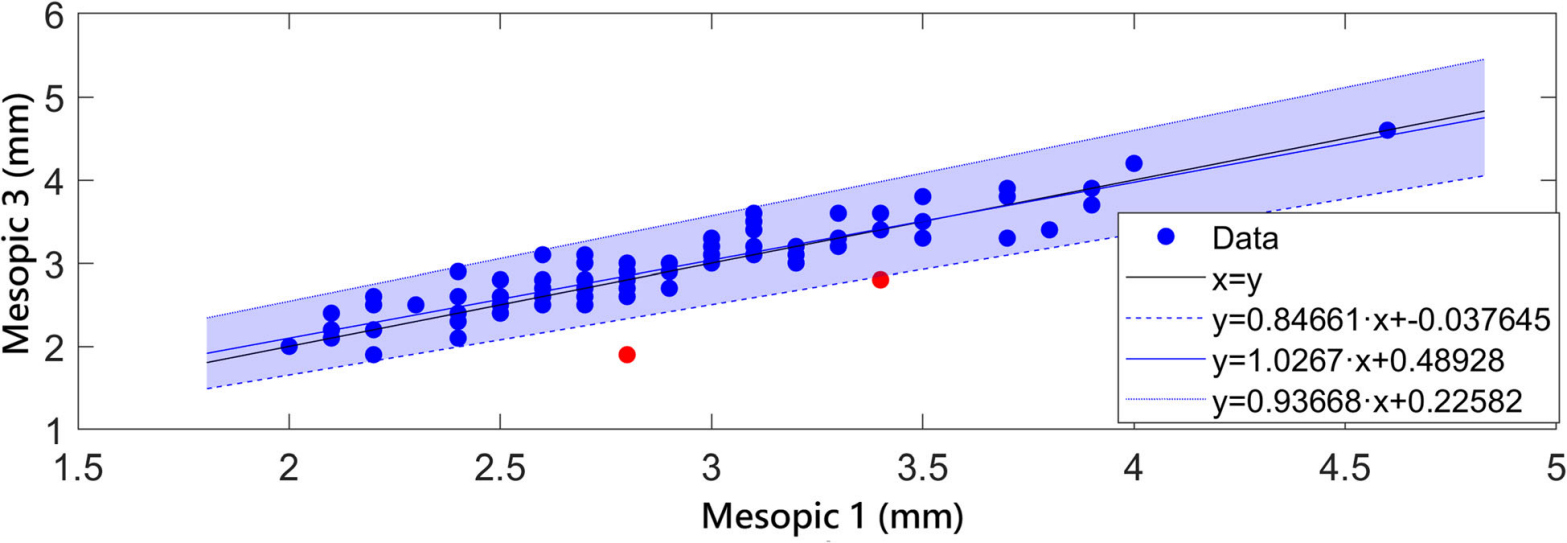
Passing-Bablok interchangeability analysis of the first and third measurements of pupil size under mesopic conditions. The confidence interval of x-intercept (coloured area) and slope of the adjusted lines includes the values of 0 and 1, respectively

Concerning the differences between repeated measures under the rest of light conditions, they were not statistically significant for L conditions (*p* = 0.751). In contrast, statistically significant differences between repeated measures were found for M and P conditions (*p* = 0.002 and *p* = 0.003, respectively). Statistically significant differences between pairs in the post-hoc analysis were only found between first and second measurements (*p* < 0.001) and also between first and third (*p* = 0.042) under M conditions. Concerning P conditions, although the *p*-value of the global comparative analysis was below 0.05 and therefore was representative of the presence of statistically significant differences, this was not observed when performing comparison by pairs after applying the Bonferroni correction. The Passing-Bablok analysis was used to evaluate the level of interchangeability between the first and second, and between the first and third pupil measurements obtained under M conditions. With this type of analysis, the two measurements compared are represented in a cartesian display and adjusted to a line. If the confidence interval associated to the x-intercept and the slope of the adjusted line include 0 and 1, respectively, the hypothesis of interchangeability cannot be rejected. In our data, the Passing-Bablok test only showed clinically relevant differences when first and second repeated measurements of M pupil size were compared (CI x-intercept: 0.10 to 0.65; CI slope: 0.80 to 0.99) (Fig. 1), but not for the comparison between first and third measurements (Fig. 2) (CI x-intercept: − 0.04 to 0.49; CI slope: 0.85 to 1.03).

### Comparative analysis between right and left eyes

In a subgroup of 75 patients from the total population, bilateral measurements of pupil diameter (mm) was performed. The results of this analysis are summarized in Table 3. As shown, median pupil values obtained under all light conditions were equal for both eyes, while mean differences in pupil size between fellow eyes were always below 0.02 mm, with a trend to obtain smaller pupils in the left eye compared to the right eye. The standard deviations of both eyes were similar, and the amplitude of ranges did not differ more than 0.2 mm. No statistically significant differences in pupil size were found between right and left eyes in any light condition evaluated (S, *p* = 0.944; L, *p* = 0.654; M, *p* = 0.227; P, *p* = 0.395). In the normally distributed parameters (S and L pupil sizes), the ICC of right and left eye measurements were calculated, obtaining values of 0.776 and 0.850 for S and L, respectively.

**Table 3:** Mean, median, standard deviation (SD), variance and range for each eye pupillometric measurement (75 patients) under the four illumination conditions evaluated. S: Scotopic level, L: Low level, M: mesopic level, P: Photopic level

	S_RE	S_LE	L_RE	L_LE	M_RE	M_LE	P_RE	P_LE
Mean	5.17	5.17	3.17	3.15	2.94	2.93	2.59	2.57
Median	5.20	5.20	3.10	3.10	2.90	2.90	2.50	2.50
SD	0.92	0.89	0.61	0.61	0.48	0.49	0.40	0.38
Variance	0.85	0.79	0.38	0.37	0.23	0.24	0.16	0.15
Range	3.90	4.00	2.80	2.90	2.50	2.70	2.30	2.10

## Discussion

Although there are some studies comparing the pupil size measurements obtained with different devices, the number of studies evaluating the consistency of this type of measurements with each device is limited. Furthermore, the sample sizes used in previous studies evaluating and comparing the pupil size measurements obtained with different methodologies are reduced [2–10], not allowing to extract consistent conclusions in most of cases. Other sources of variability between studies on pupillometry are adaptation conditions prior to the measurements, the interval of time between repeated measurements and the involvement of one or various examiners. This makes difficult the comparison of the outcomes obtained in all these studies and therefore to extract general conclusions. In the current study, the consistency of pupil size measurements obtained under different light conditions with a new system was evaluated to confirm its clinical usefulness.

The analysis of the difference in the amplitude of the range in each repeated measurement of S, L, M and P showed the presence of variations between 0.2 and 0.3 mm. This level of variability is acceptable and confirms the consistency of the pupil size measurements provided by this system. Indeed, according to previous studies, a higher level of variability was expected under photopic and mesopic conditions considering the contribution of the pupillary hippus [2]. Likewise, S_w_ was below 0.2 mm for the repeated measurements obtained under the four light conditions evaluated.

Although the maximum difference between repeated measurements found in our study was close or equal to 1 mm, most of such differences did not exceed the value of 0.5 mm (between 82% of S and 100% of P measurements below this value). These outcomes were consistent or somewhat worse than those provided by other authors with other systems and in most of cases under different light and adaptation conditions [2, 4, 9, 10]. Robl et al. [2] used a digital infrared pupillometer (Procyon P2000 SA) to evaluate mainly differences between two pupil measurements obtained in different days, but they also reported differences of 0.62 mm between repeated pupil size measures obtained during the same session under scotopic conditions. Boxer Wachler and Krueger [10] in 1999 evaluated the difference between the higher and lower pupil size measurements during the hippus cycle in 14 myopic eyes, obtaining a parameter that they called coefficient of repeatability (two times SD of measurements during the hippus cycle). They reported values for such coefficient from 0.6 to 1.4 mm using an infrared pupillometry (different light conditions and two different examiners), and from 1 to 1.2 mm using the Rosenbaum pupillometry. Furthermore, these same authors also compared in 2000 the differences in the pupil size measurements obtained with three devices using luminances that varied between 0.05 to 344 cd/m^2^: C-Scan, Masterview, and EyeMap [9]. Specifically, these authors obtained coefficients of repeatability of 0.56 mm, 0.46 mm, and 0.44 mm for the C-Scan, Masterview, and EyeMap devices, respectively.

Other authors have found differences between repeated pupil size measurements below 0.5 mm, such as Starck et al. [8]. These authors evaluated the scotopic pupil size in 16 patients comparing two measurements performed with a slit lamp-based cobalt blue light method and with an infrared video-based system. The differences between repeated measurements with the slitlamp-based method were 0.09 and 0.18 mm for two different observers [8]. Maldonado et al. [4] also evaluated the intrasession repeatability of pupil size measurements obtained with a slit lamp-based cobalt blue light method, obtaining a S_w_ of 0.7 mm and an ICC of more than 0.9. In our sample, ICC was 0.97 for S pupil size measurements, with no significant differences between repeated measurements. Likewise, with the system used, no statistically significant differences between repeated measurements were found for L. Concerning M and P, although there were globally significant differences between repeated measurements, these differences were not clinically relevant. A clinical significance level of 0.5 mm was considered, as this is the value of the standard deviation (potential variability of measurement) of the pupil size measured in a healthy population for most of illumination conditions [1–10]. Only clinically relevant differences were found for the comparison between the first and the second measurements obtained under mesopic conditions according to the Passing-Bablok analysis. This may be due to several factors including a potential higher variability due to the performance of the repeated measurements during different phases of the pupillary hippus. In spite of this trend of obtaining a clinically relevant difference between the first and second mesopic measurement, it should be considered that S_w_ for M was 0.1 mm, the maximum difference between repeated measurements was 1.0 mm and 96% of eyes showed a maximum difference of 0.5 mm or lower. One clinical recommendation that can be adopted is to take with this system three consecutive measurements under this light condition to minimize the potential variability between the first and the second measure.

Finally, as apparently isocoric patients (slit lamp examination) were included in our sample, another analysis was performed to confirm the accuracy of the pupillometric system evaluated. Specifically, interocular differences in a sample of patients evaluated bilaterally were analysed. As expected, no statistically significant differences in S, L, M and P were found between right and left eyes, with equal median values for both eyes.

## Conclusions

In conclusion, the VX120 system can provide consistent measurements of pupil size under scotopic, low mesopic and photopic conditions, with a relative limitation under mesopic conditions. When evaluating mesopic pupillary response, three consecutive measurements are recommended to be obtained to minimize the potential variability between the first and second measure. More studies evaluating the consistency of pupil size measurements in pathological conditions leading to anisocoric responses should be conducted to confirm the outcomes obtained in healthy eyes.

## Data Availability

The datasets used and/or analysed during the current study available from the corresponding author on reasonable request.

## Abbreviations

IOP: intraocular pressure
UDVA: uncorrected distance visual acuity
CDVA: corrected distance visual acuity
P: photopic pupil diameter
M: mesopic pupil diameter
L: low mesopic pupil diameter
S: scotopic pupil diameter
ANOVA: analysis of variance
ICC: intraclass correlation coefficient
S_w_: within-subject standard deviation
CI: confidence interval
SD: standard deviation
